# Study on post-traumatic stress disorder and influencing factors in adult ICU patients on mechanical ventilation using latent profile analysis: a cross-sectional survey

**DOI:** 10.3389/fpsyg.2025.1578276

**Published:** 2025-10-21

**Authors:** Yunting Li, Xiaoli Yuan, Mi Liu, Yan Xiong, Jiabi Zhang

**Affiliations:** ^1^Affiliated Hospital of Zunyi Medical University, Zunyi, China; ^2^KweiChow Moutai Hospital, Zunyi, Guizhou, China

**Keywords:** respiration, artificial, PTSD, latent profile analysis, influencing factors, nursing

## Abstract

**Objective:**

This study aimed to explore the potential classification and influencing factors of post-traumatic stress disorder (PTSD) in intensive care unit (ICU) patients receiving mechanical ventilation to provide a theoretical basis for formulating targeted intervention measures.

**Methods:**

A total of 229 patients on mechanical ventilation who were hospitalized in the intensive care unit of a Class III Grade A hospital in Zunyi from August 2023 to July 2024 were selected as research participants using a purposive sampling method. The General information questionnaire, Eysenck Personality Questionnaire Revised, Short Scale for Chinese (EPQ-RSC), Simplified Coping Style Questionnaire (SCSQ), Perceived Social Support Scale (PSSS), and Hospital Anxiety and Depression Scale (HADS) were used to assess the patients within 7 days after discharge from the ICU. One month after extubation, a cross-sectional survey was conducted using the Impact of Event Scale-Revised (IES-R). Latent profile analysis (LPA) was used to analyze the latent subtypes of PTSD, and univariate analysis and a disordered multivariate logistic regression model were used to evaluate the influencing factors associated with different types of PTSD.

**Results:**

A total of 215 valid questionnaires were collected, and the effective recovery rate was 93.89%. The incidence of PTSD was 14.9% (95% CI: 10.12%−19.64%). There were three latent categories of PTSD among the ICU patients on mechanical ventilation: the “low-stress group” (56.8%, *n* = 112), the “medium-stress group” (31.6%, *n* = 68), and the “high-stress group” (11.6%, *n* = 25). Multinomial logistic regression analysis, using the low-stress group as the reference, identified that higher educational attainment (OR [95% CI]: 1.359 [1.172–1.576], *p* < 0.001) and elevated scores on the Hospital Anxiety and Depression Scale (OR [95% CI]: 1.447 [1.186–11.767], *p* < 0.001) were statistically significant predictors of membership in the medium- and high-stress groups.

**Conclusion:**

PTSD symptoms among mechanically ventilated ICU survivors manifest in three distinct profiles. Our findings strongly recommend early psychological screening, particularly focusing on anxiety and depression levels and patients' educational background. Medical staff should formulate targeted intervention plans based on the characteristics of different patient categories to lower the level of PTSD in patients.

## 1 Introduction

Mechanical ventilation is one of the important life support methods commonly used in the field of emergency and critical care medicine. It is mainly used to support patients who cannot maintain airway patency or require improved oxygenation for various reasons. Studies have reported that approximately 30%−88% of patients treated in intensive care units (ICUs) require mechanical ventilation (Zhang et al., 2021). Receiving mechanical ventilation treatment and experiencing a prolonged ICU stay have been shown to increase the risk of post-traumatic stress disorder (PTSD; Sivanathan et al., 2019). In the process of receiving mechanical ventilation treatment, patients endure different degrees of physical and psychological discomfort, such as sputum suction, expression disorders, man-machine confrontation, and other factors. These factors cause body stress and increase the patients' sense of helplessness and fear, thereby inducing PTSD (Yanjie et al., 2019). Studies have shown that the incidence of PTSD in ICU patients on mechanical ventilation is 30.55%−34.4% (Garrouste-Orgeas et al., 2019; Xin and Qiang, 2020).

PTSD refers to the intrusive recall of the event, hyperarousal symptoms, and avoidance behaviors related to the traumatic event after experiencing a life-threatening or perceived life-threatening event (Askari Hosseini et al., 2021). PTSD patients may experience changes in the structure of the central nervous system and an increased risk of anxiety or depression (Stienen et al., 2019) and even exhibit self-harm or aggressive behaviors (Tronstad et al., 2021). The most common clinical manifestation is sleep disorders, with an incidence rate of 70%−91% (Mascarel et al., 2019).

Most previous studies, both domestic and international, have relied on scale summary scores to categorize PTSD severity, often overlooking the heterogeneity in symptom patterns among patients. For instance, several large-scale international studies have focused on predictor analyses or overall prevalence (Parker et al., 2015; Davydow et al., 2008), while similar domestic studies have primarily described risk factors and symptom distributions using conventional statistical methods (Xin and Qiang, 2020; Li, 2022). Although these studies provide valuable insights into PTSD frequency and general correlates, they do not capture distinct subgroups based on symptom profiles, limiting the potential for tailored interventions.

Current studies primarily classify the PTSD level of patients according to the score of the scale, ignoring the heterogeneity among groups. This limitation hinders the implementation of precise interventions, thereby reducing their effectiveness. Latent profile analysis (LPA) classifies research subjects into several potentially different categories based on multiple observable continuous variables, reveals potential groups within the data, and further explores the differences between different groups (Yin et al., 2020; Li et al., 2024). Therefore, this study used LPA to explore the potential profile characteristics of PTSD in ICU patients on mechanical ventilation, and a multivariate logistic regression model was used to further analyze the influencing factors of different potential categories of PTSD, aiming to provide a reference for medical staff to formulate precise and personalized intervention programs and reduce the psychological distress of patients.

## 2 Methods

### 2.1 Respondents

A total of 229 patients on mechanical ventilation who were hospitalized in the Department of Critical Care Medicine of a Class III Grade A hospital in Zunyi from August 2023 to July 2024 were selected as research participants. The inclusion criteria were as follows: ① Invasive mechanical ventilation for ≥48 h; ② age ≥18 years; ③ No major stressful events within 1 year before admission; ④ clear consciousness and normal communication skills; and ⑤ informed consent provided by patients and their families, with voluntary participation. The exclusion criteria were as follows: ① Patients with a history of mental illness or those using psychotropic drugs and ② patients who returned to the ICU and were intubated during the investigation period. The dropout criteria were as follows: ① Patients who died or experienced deterioration during the investigation; ② patients who voluntarily withdrew or did not cooperate; and ③ failure to contact the patient for 3 times on different days. The sample size was calculated based on the sample size requirements of the latent profile analysis. A total of 23 variables were included in this study, and the sample size was 5–10 times the number of variables (Ni and Chen, 2010). Considering a loss rate of 20%, the required sample size was estimated to be between 138 and 276 cases, and 215 cases were finally included. This study was approved by the ethics committee of the hospital (KLLY-2023-055). All participants provided informed consent and participated voluntarily.

### 2.2 Survey tools

#### 2.2.1 General information questionnaire

The questionnaire was self-designed by the researchers based on a literature review. It included a total of 19 items covering demographic and clinical information such as gender, age, marital status, education level, place of residence, occupational status, swallowing, smoking and drinking habits, hospitalization expenses, payment methods for medical costs, APACHE II score, duration of mechanical ventilation, total length of hospital stay, length of ICU stay, time from extubation to ICU discharge, hypertension, diabetes, coronary heart disease, and number of chronic diseases. Demographic data were filled in by patients or their family members, and clinical data were obtained from the hospital's electronic medical record system or by consulting doctors.

#### 2.2.2 Impact of event scale-revised (IES-R)

The scale was developed by Weiss et al. in 1997 and later sinicized by Huang et al. (Huang et al., 2006), the scale consists of 22 items across three dimensions: intrusive thinking symptoms (eight items), avoidance symptoms (eight items), and hyperarousal symptoms (six items). It uses a Five-point Likert scale ranging from 0 (“never”) to 4 (“always”). The total score ranges from 0 to 88. Each item was scored by the patient according to the impact of a traumatic event experienced in the past 7 days. The cut-off value was set at 35 points; a total score of ≥35 points indicated positive PTSD symptoms. A total score less than 35 points indicated negative PTSD symptoms. It is important to note that the IES-R serves as a screening tool for PTSD symptoms and is not a diagnostic instrument. The scale demonstrates good internal consistency, with a total Cronbach's α of 0.89, and the Cronbach's α coefficients for the three dimensions were 0.77, 0.83, and 0.76, respectively.

#### 2.2.3 Eysenck personality questionnaire revised, short scale for Chinese (EPQ-RSC)

Developed by British psychologist Eysenck et al., the Chinese version was introduced by Qian et al. (2000). The scale includes four subscales: P (mental quality scale), E (extraversion scale), N (neuroticism scale), and L (camouflage scale). Each subscale contains 12 items and is scored from 0 to 12, with higher scores indicating more intense personality traits. The Cronbach's α coefficients for the E, N, and L subscales of the EPQ-RSC ranged from 0.74 to 0.78, and the Cronbach's α coefficients for the P subscale ranged from 0.54 to 0.60. Two personality traits, E and N, were analyzed in this study.

#### 2.2.4 Simplified coping style questionnaire (SCSQ)

The questionnaire was developed by Xie (1998) based on the coping style scale in foreign countries and adapted to the characteristics of the Chinese population. It is widely used in the field of domestic psychology. There are 20 items in total, including positive coping (items 1–12) and negative coping (items 13–20). A Four-point Likert scale is used, with scores ranging from 0 (“do not use”) to 3 (“often use”), and the results yield separate scores for positive coping styles and negative coping styles. The Cronbach's α coefficient for the full scale was 0.9, with the positive coping subscale at 0.89 and the negative coping subscale at 0.78.

#### 2.2.5 Perceived social support scale (PSSS)

The scale was developed by Zimmer et al., in 1988, and translated into Chinese by Jiang et al. (Zimet et al., 1988). It includes three dimensions—family support, other support (such as leaders, relatives, and colleagues), and friend support—with a total of 12 items. It uses a Seven-point Likert scale, ranging from 1 (“strongly disagree”) to 7 (“strongly agree”). The total score reflects the level of social support and is categorized into three levels: low support, medium support, and high support. The levels are 12–36 points (low support), 37–60 points (medium support), and 61–84 points (high support). The total score is proportional to the level of social support. The total Cronbach's α for the scale was 0.92, and the Cronbach's α coefficients for the three subscales were 0.91, 0.89, and 0.81, respectively.

#### 2.2.6 Hospital anxiety and depression scale (HADS)

The scale was developed by Zigmond and Snaith (1983), and it consists of two subscales: Anxiety (A) and Depression (D), each with seven items. A Four-point Likert scale is used, with scores ranging from 0 to 3 for each item. The total score for each subscale ranges from 0 to 21 points. A score of 8 or higher on either the Anxiety (A) or Depression (D) subscale indicates a positive screen for anxiety or depression. Zheng et al. (2003) showed that the Cronbach's α coefficients for the Anxiety and Depression subscales were 0.806 and 0.791, respectively, indicating that the scale has good reliability and validity.

### 2.3 Methods of data collection and quality control

The investigators strictly screened the participants according to the inclusion and exclusion criteria, fully explained the purpose, significance, and questionnaire instructions of the study, and obtained the participants' informed consent before collecting basic information within 7 days after they were transferred out of the ICU. At the same time, the researchers made an appointment with the participants for the next follow-up visit and collected data through face-to-face visits, telephone calls, WeChat, and other methods. During the questionnaire completion process, if the patients had any questions, standardized statements were used to answer them. If the patient had difficulty with reading or writing, after obtaining their consent, the investigator repeated the questions in a neutral tone and checked with them. All questionnaires were collected on site, and two members of the research team entered and cross-checked the data in pairs to identify any missing items and excluded questionnaires with regular responses.

### 2.4 Statistical methods

Mplus 8.3 was used to establish a potential profile model. Starting from a single category model, the number of categories was gradually increased, and the best-fitting model was selected according to the model adaptation index and clinical significance. Model adaptation indicators included the Akaike information criterion (AIC), Bayesian information criterion (BIC), and adjusted BIC (aBIC). The smaller the statistical value, the better the model fitting effect. A statistically significant p-value (*p* < 0.05) for the Bootstrap Likelihood Ratio Test (BLRT) and the Lo-Mendell-Rubin (LMR) test allows for the rejection of the null hypothesis, supporting that the *k*-class model fits the data significantly better than the (*k*-1)-class model (Wang et al., 2017). The entropy index was used to evaluate the accuracy of the classification. The value ranges from 0 to 1, with values closer to 1 indicating more accurate classification. When an entropy value is ≥0.8, the accuracy rate of classification is ≥90% (Mengcheng et al., 2017). SPSS 29.0 was used for data analysis. Count data were described using frequency and percentage, and the χ^2^ test was used for comparisons between the groups. Measurement data with a normal distribution were expressed as $\bar{x}\;\pm \;s$, and one-way analysis of variance (ANOVA) was used for comparisons between the groups. Multinomial logistic regression analysis was used to examine the influencing factors across multiple groups. A *p*-value of < 0.05 was considered to be statistically significant.

## 3 Results

### 3.1 General information of the survey respondents

**Figure 1:**
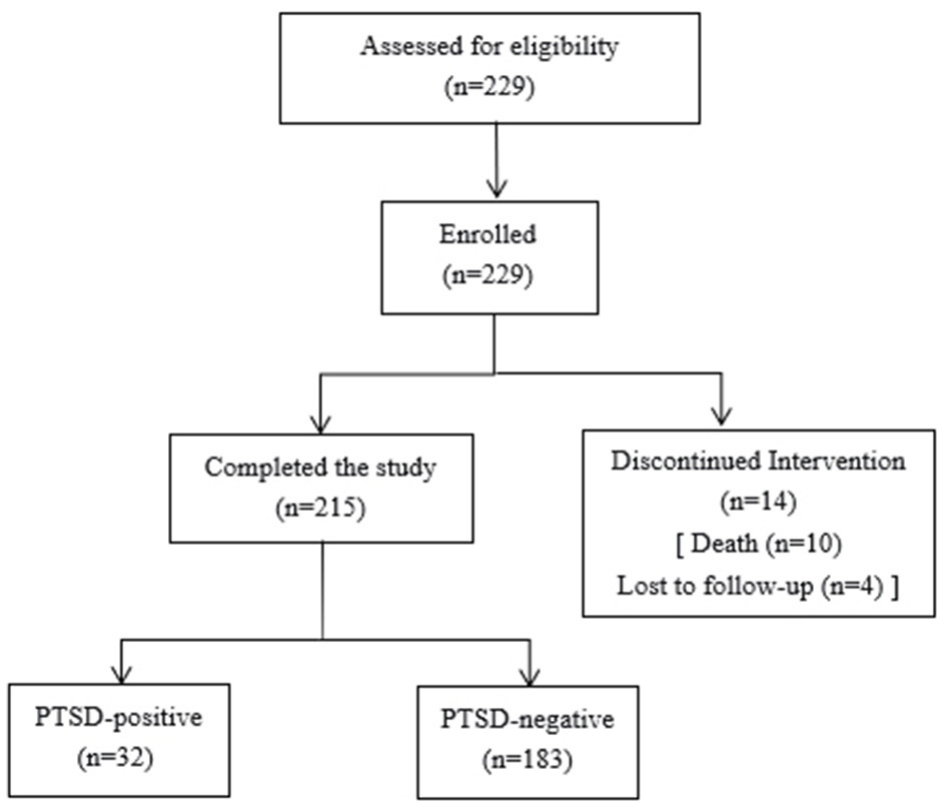
CONSORT flow diagram.

A total of 229 questionnaires were distributed in this study, and 215 effective questionnaires were obtained by excluding 10 dead patients and patients who were lost to follow-up, with an effective questionnaire recovery rate of 93.89%. Figure 1 shows the CONSORT flow diagram of participant enrollment, allocation, and follow-up. The 215 patients on mechanical ventilation in the ICU had a mean age of 56.67 ± 14.69 years. There were 133 male (61.9%) and 82 female participants (38.1%). In terms of education level, 98 (45.6%) had completed primary school or below, 74 (34.4%) had a junior high school education, and 43 (20%) had a senior high school education or above. A total of 188 participants were married (87.4%), nine were unmarried (4.2%), four were divorced (1.9%), and 14 were widowed (6.5%). Regarding occupational status, 63 (29.3%) were unemployed, 29 (13.5%) were employed, 57 (26.5%) were self-employed, 49 (22.8%) were farmers, 14 (6.5%) were retired, and three (1.4%) were students. A total of 52 participants (24.3%) lived in urban areas, while 163 (75.8%) lived in rural areas. The medical payment methods included 156 using urban residents' medical insurance (72.6%), 34 using urban workers' medical insurance (15.8%), 24 using fully out-of-pocket (11.2%), and one using commercial medical insurance (0.4%). In total, 83 participants were smokers (38.6%) and 132 were non-smokers (61.4%); 64 (29.8%) consumed alcohol, while 151 (70.2%) did not consume alcohol. A total of 78 (36.3%) patients had hypertension, while 137 (63.7%) did not. There were 36 patients (16.7%) with diabetes and 179 patients (83.3%) without diabetes. There were seven patients (3.3%) with coronary heart disease and 208 patients (96.7%) without coronary heart disease. The number of chronic diseases per patient was as follows: 92 patients (42.8%) had none, 71 (33.0%) had one, 37 (17.2%) had two, and 15 (7.0%) had more than three. Among the 215 patients in this study, the majority (69.8%, *n* = 150) required mechanical ventilation for ≤ 7 days, while 30.2% (*n* = 65) required it for >7 days. The total hospital stay was ≤ 14 days for 23.7% (*n* = 51) of patients, 15–28 days for 42.8% (*n* = 92), and ≥29 days for 33.5% (*n* = 72). Regarding ICU stay, 42.8% (*n* = 92) were hospitalized in the ICU for ≤ 7 days, 40.5% (*n* = 87) for 8–13 days, and 16.7% (*n* = 36) for ≥14 days. The duration from extubation to ICU transfer was 95.03 ± 81.43 h. The APACHE II score was 21.65 ± 4.78. The hospitalization cost was 87501.35 ± 58231.29 yuan.

### 3.2 Status of PTSD in the patients on mechanical ventilation in the ICU

The total IES-R score among the ICU patients on mechanical ventilation was 20.53 ± 12.57. The scores for each symptom dimension were as follows: 6.45 ± 4.83 for intrusive thinking, 7.90 ± 5.35 for avoidance symptoms, and 6.18 ± 3.48 for hyperarousal symptoms. In this study, 32 patients on mechanical ventilation developed PTSD, with an incidence rate of 14.9% (95%CI: 10.12%−19.64%).

### 3.3 Latent profile analysis of PTSD in the ICU patients on mechanical ventilation

The latent profile model was fitted based on the IES-R scale scores from the mechanically ventilated ICU patients. A series of five models (ranging from one to five classes) was estimated and compared (Table 1). Although the AIC, BIC, and aBIC values continued to decrease as the number of classes increased, the Lo–Mendell–Rubin likelihood ratio test (LMRT) for the three-class model was not statistically significant (*p* > 0.05). However, the bootstrap likelihood ratio test (BLRT) for the three-class model was significant (*p* < 0.05), indicating a superior fit compared to the two-class model. Furthermore, the three-class model demonstrated high classification accuracy, as evidenced by an entropy value of 0.964, which exceeded the recommended threshold of 0.8. Most importantly, the three-class solution offered the most clinically meaningful and interpretable profile differentiation, distinguishing patients with low, medium, and high symptom severity in a manner consistent with established clinical understanding. Therefore, based on the significant BLRT result, high entropy, and clinical interpretability, the three-class model was selected as the optimal solution. The accuracy of this model was further validated using discriminant analysis, which yielded a posterior probability of 99.1%, confirming its reliability and high discriminative power.

**Table 1:** Fitting information of the latent profile model for PTSD in the ICU patients on mechanical ventilation (*n* = 215).

**Model**	**AIC**	**BIC**	**aBIC**	***P***		**Entropy**	**Class probability (%)**
				**LMRT**	**BLRT**		
1	11890.66	12038.97	11899.54	–	–	–	100
2	10185.32	10411.15	10198.84	< 0.001	< 0.001	0.975	67.2/32.8
3	9837.61	10140.96	9855.77	0.4432	< 0.001	0.964	56.7/31.9/11.4
4	9678.94	10059.83	9701.75	0.4334	< 0.001	0.937	45.5/21.5/24.3/8.7
5	9597.44	10055.85	9624.89	0.7598	< 0.001	0.947	44.6/23.5/5.7/18/8.2

AIC, Akaike information criterion; BIC, Bayesian information criterion; aBIC, the Bayesian information criterion for sample correction; LMRT, Lo–Mendell–Rubin likelihood ratio test; BLRT, bootstrap likelihood ratio test.

**Figure 2:**
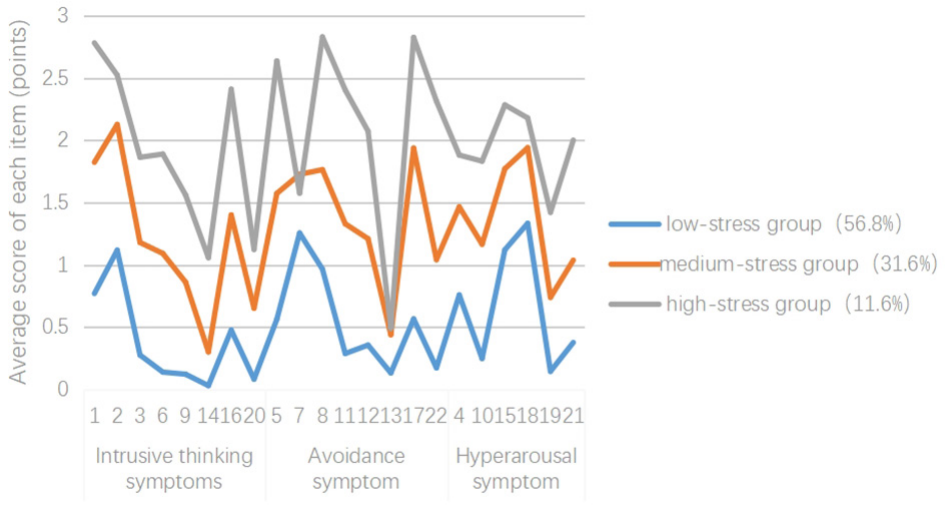
Characteristic distribution of the three potential PTSD types in the patients on mechanical ventilation in the ICU.

Based on model 3, the mean scores for the categories across the three dimensions of the IES-R scale are shown in Figure 2. According to the fluctuation of the average line chart of each item in the latent profile, the characteristics and attributes of the three latent subtypes of PTSD were analyzed and named. The total IES-R score for type 1 was 11.3 ± 4.83, indicating a generally low level. The total and dimension scores for type 1 were lower than those for type 2 and type 3. Therefore, type 1 was labeled the “low stress group,” with a total of 112 individuals (56.8%). The total score for type 2 was 28.56 ± 5.31, indicating a moderate level. Therefore, type 2 was named as the “medium stress group,” comprising 68 students (31.6%). The total score for type 3 was 43.72 ± 6.12, and this total score was the highest. Therefore, type 3 was labeled the “high stress group,” comprising 25 cases (11.6%).

### 3.4 Univariate analysis of the potential categories of PTSD in the mechanically ventilated ICU patients

Univariate analyses were conducted to examine the differences in demographic and clinical characteristics across the three identified latent profiles. Categorical variables (e.g., education level) were compared using the chi-squared test. Continuous variables were assessed for normality using the Shapiro–Wilk test. Based on the distribution, normally distributed data (e.g., PSSS score and HADS scores) were compared using one-way analysis of variance (ANOVA), while non-normally distributed data (e.g., hospitalization expenses and duration from extubation to ICU discharge) were compared using the Kruskal–Wallis test. The results indicated statistically significant differences among the three groups in education level, hospitalization expenses, duration from extubation to ICU discharge, HADS-A score, HADS-D score, PSSS score, and SCSQ positive response score (all *p* < 0.05), as detailed in Table 2.

**Table 2:** Univariate analysis of the potential categories of PTSD in the mechanically ventilated patients in the ICU (*n* = 215).

**Items**		**Low stress group (*n* = 122)**	**Medium stress group (*n* = 68)**	**High stress group (*n* = 25)**	**Test statistic**	***P***
Level of education [name (percentage, %)]	Primary school degree and below	42 (34.5)	37 (54.4)	19 (76)	19.548^a^	0.001
	Junior high school degree	47 (38.5)	22 (32.4)	5 (20)		
	High school or above	33 (27)	9 (13.2)	1 (4)		
Cost of hospitalization (yuan, $\bar{x}$ ±*s*)		87501.35 ± 58231.29			10.363^b^	0.006
Duration from extubation to ICU discharge (hours, $\bar{x}$ ±*s*)		95.03 ± 81.43			3.491^c^	0.032
PSSS score (points, $\bar{x}$ ±*s*)		60.04 ± 10.40			3.348^c^	0.037
HADS score (points, $\bar{x}$ ±*s*)	HADS-A	9.19 ± 4.07			37.685^c^	< 0.001
	HADS-D	7.96 ± 4.19			16.356^c^	< 0.001
SCSQ score (points, $\bar{x}$ ±*s*)	Positive response	20.92 ± 4.99			6.098^b^	0.047
	Negative response	10.80 ± 3.57			1.103^b^	0.576
ERQ-RSC score (points, $\bar{x}$ ±*s*)	Extraversion	8.81 ± 3.13			2.963^b^	0.227
	Neuroticism	4.33 ± 2.91			15.088^b^	0.001

Only statistically significant data are presented in this table. ^a^χ^2^ value; ^b^H value; ^c^F value. PSSS, Perceived Social Support Scale; HADS, Hospital Anxiety and Depression Scale; HADS-A, Hospital Anxiety and Depression Scale—Anxiety subscale; HADS-D, Hospital Anxiety and Depression Scale—Depression subscale; SCSQ, Simplified Coping Style Questionnaire; ERQ-RSC, Eysenck Personality Questionnaire Revised, Short Scale for Chinese.

### 3.5 Multivariate analysis of the potential categories of PTSD in the mechanically ventilated ICU patients

To identify independent predictors of profile membership, variables showing significance in univariate analyses (*p* < 0.05) were included as independent variables in a multinomial logistic regression model, with the three-category PTSD profile serving as the dependent variable. Continuous variables were analyzed without transformation, and categorical variables were dummy-coded, as specified in Table 3.

**Table 3:** Variable assignment methods.

**Variables**	**Assignment of value**		
Latent subtypes of PTSD	Low stress group = 1	Medium stress group = 2	High stress group = 3
Degree of education	Primary school degree and below = 1	Junior high school degree = 2	High school or above = 3

The analysis revealed that higher HADS-A scores and lower educational attainment were significant independent predictors of profile membership.

Compared to the low stress group, the patients with higher HADS-A scores (OR = 1.375, 95% CI: 1.184–1.597, *p* < 0.001) and those with a primary school education or below (OR = 3.605, 95% CI: 1.281–10.142, *p* = 0.015) were significantly more likely to belong to the medium stress group.

Similarly, compared to the low stress group, patients with higher HADS-A scores (OR = 1.489, 95% CI: 1.212–1.829, *p* < 0.001) and those with a primary school education or below (OR = 16.268, 95% CI: 1.785–148.241, *p* = 0.013) were markedly more likely to belong to the high stress group (see Table 4).

**Table 4:** Multiple logistic regression analysis of the potential categories of PTSD in the patients on mechanical ventilation in the ICU (*n* = 215).

**Items**	**β**	**SE**	**Wald χ^2^**	***P***	**OR**	**95% CI**
**C1 vs. C2** ^a^						
HADS-A	0.319	0.076	17.410	< 0.001	1.375	1.184–1.597
Primary school degree and below	1.282	0.528	5.902	0.015	3.605	1.281–10.142
**C1 vs. C3** ^a^						
HADS-A	0.398	0.105	14.379	< 0.001	1.489	1.212–1.829
Primary school degree and below	2.789	1.127	6.121	0.013	16.268	1.785–148.241

C1, low stress group; C2, medium stress group; C3, high stress group; ^a^C1 was used as the reference group; HADS-A, Hospital Anxiety and Depression Scale—Anxiety subscale.

To further elucidate the relationships between key continuous variables, Pearson correlation analysis was performed. A significant positive correlation was observed between HADS-A scores and PTSD symptom severity (*r* = 0.566, *p* < 0.001), indicating that higher anxiety levels were strongly associated with more severe PTSD symptoms (see Table 5). These results collectively underscore the critical role of acute anxiety and socioeconomic factors in predicting PTSD risk stratification among mechanically ventilated ICU patients.

**Table 5:** Pearson correlations between key variables.

**Variables**	**HADS-A**	**IES-R**
HADS-A	1	0.566^**^
IES-R	0.566^**^	1

^**^At the 0.01 level (two-tailed), the correlation is significant.

HADS-A, Hospital Anxiety and Depression Scale—Anxiety subscale; IES-R, Impact of Event Scale-Revised.

## 4 Discussion

### 4.1 Mechanically ventilated ICU patients have a high incidence of PTSD, which can be divided into three categories

The results of this study showed that the IES-R score of the ICU patients on mechanical ventilation was 20.53 ± 12.57, with 32 patients (14.9%) developing PTSD—a rate much higher than the 3.9% incidence in the general population (Koenen et al., 2017). ICU patients with more serious illness tend to experience faster disease progression. Due to poor immunity, they often require protective isolation measures, which greatly limit the family care time. In the process of receiving mechanical ventilation treatment, patients not only bear physical pain but also experience different degrees of psychological pressure. Due to language barriers and other factors, patients cannot get effective psychological support in time, which increases the risk of PTSD. Therefore, medical personnel should pay attention to the psychological status of patients on mechanical ventilation and make comprehensive assessment, identify PTSD symptoms early, and timely adopt multi-factor comprehensive interventions, such as targeted psychological support, health education, mindfulness therapy (Boyd and Lanius, 2018), and virtual reality technology (Wiebe et al., 2022), to prevent or slow down the occurrence and development of PTSD.

In this study, the PTSD patients on mechanical ventilation in the ICU were classified into three potential categories according to their individual characteristics: the “low stress group,” “medium stress group,” and “high stress group.” Each evaluation index indicated that the model fit was good, suggesting that there were significant individual differences among the PTSD patients on mechanical ventilation in the ICU. (1) The “low stress group” accounted for 56.8% of the patients, and the level of PTSD in the patients in this category was low, which may be attributed to the relatively high education level of patients in this category and their higher understanding and acceptance in the face of disease changes or treatment suggestions. Meanwhile, patients with higher education levels could further educate themselves about the disease through the Internet and other channels, enhancing their confidence in recovery and reducing the risk of PTSD. (2) The “medium stress group” accounted for 31.6% of the participants, and the PTSD level of the patients in this category was generally moderate, but the avoidance dimension scores were high. The reason may be that these patients, having just passed the critical life-threatening period, are unwilling to recall related events. Persistent avoidance of fear and other adverse psychological conditions may continue to develop, damaging their mental health and social life. (3) The “high stress group” accounted for 11.6% of the patients, and the level of PTSD in this category of patients was higher, and the scores for intrusive thinking symptoms and hyperarousal symptoms were significantly higher. This may be because, compared to the other categories, this category had a higher proportion of patients with lower education levels and elevated anxiety. Anxiety leads to disturbances in the sleep–wake cycle, resulting in fatigue and high alertness, which further increases the risk of PTSD (Jakšić et al., 2012). In addition, a previous study (Xin and Qiang, 2020; Li, 2022) found that a mechanical ventilation duration of >7 days and a high APACHE II score were significantly correlated with PTSD and could positively predict the risk of PTSD. However, these variables were not included in the final logistic regression equation in this study, which may be related to the small sample size of the “high stress group,” and the sample size can be expanded for further verification in future studies.

### 4.2 Identifying high-risk patients: the roles of anxiety and education in PTSD stratification

The mental health of ICU patients has attracted widespread attention from scholars both domestically and internationally, making the development of precise clinical intervention measures an urgent priority. This study employed multivariate logistic regression analysis to explore independent predictors influencing the potential subtypes of PTSD in mechanically ventilated ICU patients, providing an empirical basis for the early identification of high-risk groups and the implementation of targeted interventions.

The results indicated that anxiety symptoms (HADS-A score) and education level are core predictors distinguishing the “low stress group” from the “medium and high stress groups.” Compared to the low stress group, both the medium stress group (OR = 1.375, 95% CI: 1.184–1.597, *p* < 0.001) and the high stress group (OR = 1.489, 95% CI: 1.212–1.829, *p* < 0.001) exhibited significantly higher levels of anxiety, consistent with previous research findings (Xin and Qiang, 2020; Li et al., 2023). Anxiety is not only a comorbidity of PTSD but also a critical driver of its development and progression (Navarra-Ventura et al., 2024; Hatch et al., 2018). A potential mechanism is that anxiety heightens neurotransmitter activity, which increases patients' sensitivity to physical discomfort (Carpenter and Bragdon, 2022), thereby amplifying psychological burden and elevating the risk of PTSD.

Education level also significantly influenced PTSD classification. Using “high school or above” as the reference, the patients with a “primary school or below” education level had a significantly higher risk of belonging to the medium stress group (OR = 3.605, *p* < 0.05) and the high stress group (OR = 16.268, *p* < 0.05). Education level often reflects an individual's health literacy, coping resources, and socioeconomic status. Patients with lower education levels may be more susceptible to severe stress reactions due to limited health information comprehension, inadequate coping strategies, and unequal access to medical resources. This finding aligns with the results reported by Li et al. (2023). Lower cognitive function has been identified as a risk factor for PTSD, and since education is highly correlated with cognitive ability, it serves as an important social determinant in protecting against PTSD and other psychiatric disorders (Sayed and Iacoviello, 2015).

It is noteworthy that, although anxiety and education level effectively differentiated low-stress states, the multivariate logistic regression model in this study did not identify any variables that could reliably distinguish between the “medium stress group” and the “high stress group” (all *p* > 0.05). This result is likely attributable to the relatively small sample size of the high stress group in this study. This limitation in statistical power may have prevented clinically meaningful differences from reaching statistical significance. Furthermore, patients with “medium stress” and “high stress” may share more similarities in demographic factors, clinical baseline characteristics, and most psychological measures. Distinguishing between these two groups may require exploring other factors not included in this study, such as genetic susceptibility, prior trauma history, or specific ICU experiences (e.g., duration of delirium, depth of sedation).

Correspondingly, this study also found that depressive symptoms, personality traits, social support, coping styles, and clinical indicators (such as hospitalization costs and time from extubation to ICU discharge) did not independently predict PTSD classification. The lack of significant differences in depressive scores across the subtypes may be because the HADS questionnaire was administered within 7 days after ICU discharge. During this period, patients typically receive substantial social support, are more focused on disease progression, and have a stronger desire to express themselves, potentially leading to a lower incidence of depression. Future studies could reassess depressive symptoms when evaluating PTSD to further validate this finding.

Based on these findings, we propose the following clinical recommendations: First, routine anxiety screening should be implemented early after ICU discharge, with focused assessment of patients with lower education levels to enable early identification of individuals at high risk of progressing from mild to more severe symptoms. Second, a stratified intervention strategy should be developed to provide enhanced psychological support for patients with high anxiety and low education levels. This could include anxiety management programs based on cognitive-behavioral therapy and disease education delivered through easily understandable materials to improve intervention accessibility and compliance. In addition, psychosocial interventions, such as mindfulness-based stress reduction and positive self-disclosure, could help patients cultivate a positive mindset and alleviate psychological pressure, thereby mitigating the progression of PTSD.

## 5 Summary

Based on the latent profile analysis, this study identified three potential categories of PTSD among the ICU patients on mechanical ventilation, with the highest proportion of patients belonging to the low stress group. At the same time, univariate analysis and multivariate analysis were used to analyze the internal and external influencing factors of different potential categories. It was found that education level, hospitalization cost, duration from extubation to ICU transfer, HADS-A score, HADS-D score, PSSS score, SCSQ score, and ERQ-RSC score all affected the level of PTSD. It is critical to emphasize that this study identified probable PTSD symptoms using the IES-R screening tool, rather than formal clinical diagnoses based on the DSM-5/ICD-11 criteria. Since functional impairment and the exclusion of other causes were not assessed, these results reflect symptomatic burden rather than confirmed diagnoses. Medical staff should identify the types and characteristics of PTSD in patients on mechanical ventilation, assess their psychological status and needs, and provide patient-centered personalized interventions to promote positive physical and mental adjustment, thereby improving their quality of life. This study could not explain the causal relationship between the factors, and the cross-sectional investigations could not reflect the trajectory of PTSD during the development of the disease. It is suggested that in the future, longitudinal studies can be conducted to analyze the dynamic interactions between PTSD and predictive factors. In addition, high-quality randomized controlled trials can be conducted to explore personalized intervention plans, further improve the formulation of precise interventions, and provide a theoretical basis for improving the quality of care for patients on mechanical ventilation in the ICU.

## Data Availability

The raw data supporting the conclusions of this article will be made available by the authors, without undue reservation.
